# Recent Animal Models of Alcoholism

**Published:** 2000

**Authors:** Rainer Spanagel

**Affiliations:** Rainer Spanagel, Ph.D., is head of the Department of Psychopharmacology, Central Institute of Mental Health, University of Heidelberg, Mannheim, Germany

**Keywords:** animal model, trend, research, AOD (alcohol or other drug) preference, AODD (AOD use disorder), relapse, AOD craving, AOD abstinence, anti-alcohol-craving agents, AOD-seeking behavior

## Abstract

Animal models on alcohol preference have a long-standing tradition in biomedical research on alcoholism. However, these models allow only limited conclusions regarding alcohol addiction. Therefore, during the past 15 years, researchers have developed new animal models that mimic different aspects of human alcohol addiction, such as craving, relapse, and loss of control over drinking. These models include the reinstatement model, the alcohol deprivation model, and the point-of-no-return model. Some of these models have been pharmacologically validated with anticraving compounds that are used clinically for treating alcoholics. The detailed behavioral characterization of these new models and their pharmacological validation also allow researchers to study the neurochemical and molecular bases of addictive behavior.

Researchers have known since 1940 that some rodents voluntarily consume alcohol in a laboratory setting.^1^ One can also assume that voluntary alcohol consumption by rodents and other mammals occurs in the wild, because some mammals, including rodents, occasionally consume large amounts of rotten fruits and exhibit abnormal behavioral patterns that may result from intoxication. Consequently, voluntary alcohol consumption, which is often observed in combination with palatable food or fluid intake, can be considered a part of the normal behavioral repertoire of rodents. These observations position rats and mice as ideal subjects for studying various aspects of human alcohol use, including alcohol reinforcement.^2^

One commonly used approach to modeling human alcohol consumption in rodents are alcohol preference studies, in which the animals are given a choice between water and alcohol solutions and the investigators measure the amount consumed of each fluid. In comparison to other behavioral studies (e.g., anxiety tests), data on alcohol consumption levels obtained by such alcohol preference experiments show little variation, even when conducted in different laboratories (Crabbe et al. 1999) and different settings. Moreover, because alcohol reinforcement is mediated by brain structures that have been strongly conserved during evolution (i.e., subcortical structures), rodent studies have an enormous potential for further elucidating the neurobiological basis of alcohol consumption and alcohol reinforcement processes in humans.

This article presents several rodent models that have been used in recent years to study various aspects of alcohol addiction. The article first reviews traditional alcohol preference models and their limitations. It then describes newer models aimed at helping researchers investigate the rodent equivalent of complex human behaviors, such as craving, relapse, and loss of control over drinking. These models have been validated in pharmacological studies and have provided some insight into the neurochemical and cellular changes underlying addictive behaviors.

## Alcohol Preference Models

As mentioned previously, researchers have conducted numerous alcohol preference studies in which the animals were offered a free choice between water and alcohol solutions of various concentrations. These studies found that when offered low alcohol concentrations (i.e., up to 6 percent weight/volume), which have a “sweet” taste, rats and mice generally drink more alcohol than water. At higher alcohol concentrations, however, at which the taste of the solution usually is aversive to rodents, large differences exist among individuals and among strains in alcohol preference. These observations suggest that animals primarily prefer alcohol because of such factors as taste, rather than because of its stimulatory effect on the central nervous system. Only a few animals exhibit an alcohol preference that results from alcohol’s pharmacological (e.g., reinforcing) effects.

The large variability in alcohol preference among individual animals and strains has allowed researchers to selectively breed rats for differential alcohol preference, generating pairs of animal strains that are characterized by particularly low or high alcohol consumption levels. The best studied pairs of lines were generated in Finland, the United States, and Sardinia. The Finnish model—called Alko Alcohol (AA) and Alko Nonalcohol (ANA) rats—comprises two strains of albino rats that based on their selection or rejection of a 10-percent alcohol solution and water, were selectively bred starting in 1963 (Eriksson 1968). The alcohol-preferring (P) rats, originally bred in Indiana, voluntarily consume 5–8 grams of alcohol per kilogram of body weight per day (g/kg/day), attaining blood alcohol concentrations of 50–200 mg/100 mL, whereas the non-alcohol-preferring rats (NP) consume less than 0.5 g/kg/day alcohol (McBride and Li 1998). The Sardinian alcohol-preferring (sP) rats also have been selectively bred for high alcohol preference and consumption for more than 20 years (Colombo 1997). These models have been used as a tool for characterizing the behavioral, neurochemical, and molecular correlates of differential voluntary alcohol consumption and preference.

A major limitation of these models is that alcohol preference alone does not necessarily indicate addictive behavior but often reflects controlled alcohol consumption. For example, animals from an alcohol-preferring strain of inbred mice called C57BL/6 have a high alcohol preference but do not meet important criteria of addictive behavior, such as loss of control over drinking. Thus, the animals’ alcohol intake decreases dramatically when they are offered diets augmented with sugar. Furthermore, the close correlation of food and alcohol consumption and the occurrence of alcohol consumption at normal times in the circadian cycle demonstrate that alcohol intake in C57BL/6 mice is controlled by normal behaviors (Dole et al. 1985). Consequently, the usefulness of alcohol-preferring inbred mouse strains as valid animal models of alcoholism is questionable.

Nevertheless, the aforementioned alcohol-preferring rat lines have allowed researchers to study numerous aspects of alcohol’s effects and their role in alcohol use. For example, studies have demonstrated clearly that these animals maintain voluntary alcohol consumption even in the presence of other palatable solutions (e.g., Lankford et al. 1991). Morever, the alcohol-preferring rats find alcohol reinforcing, because they will orally self-administer alcohol even if they have to perform a task to obtain the alcohol (i.e., under operant conditions). Finally, an elegant set of experiments has shown that P-rats consume alcohol for its reinforcing actions on the central nervous system. In those studies, the animals self-administered small amounts of alcohol via a special infusion device directly into a brain region called the ventral tegmental area (Gatto et al. 1994; Rodd-Henricks et al. 2000*b*). This brain site is critically involved in initiating the reinforcing effects of drugs of abuse.

## The Reinstatement Model

The main criterion of alcohol dependence is loss of control over drinking. Compulsive, uncontrolled alcohol-seeking and alcohol-taking behavior can occur even after long periods of abstinence and is usually associated with craving and relapse. Accordingly, animal models that measure relapse behaviors may allow researchers to investigate aspects of human alcohol dependence that are not easily addressed by preference models. One approach for measuring craving and relapse behaviors in animals is the reinstatement model (Stewart and de Wit 1987).

In a typical reinstatement experiment a rat is initially trained to press a lever for receiving alcohol or another drug. After the rat has learned this specific task, the drug is withheld, even if the animal presses the lever. After a while the rat stops pressing the lever, indicating that the lever-pressing behavior has become extinguished. Following extinction, investigators present various stimuli and assess whether these stimuli reinstate the drug-seeking behavior—that is, if they cause renewed lever responding even if the animal does not receive the drug. At least three types of stimuli can reinstate responding: (1) injection of a small dose of the drug (i.e., drug priming), (2) stress, and (3) conditioned stimuli that were previously paired during the initial training session with the delivery of the drug.

Although reinstatement models of intravenous self-administration of psychostimulants and opioids have existed for many years, few attempts have been made to transfer this paradigm to the alcohol field. Chiamulera and colleagues (1995) reported the first alcohol reinstatement study in rats. In that study, rats were trained over several months to press a lever in order to receive alcohol. After stable lever pressing was obtained, the rats were tested in extinction, receiving water instead of alcohol following lever pressing. After 8 to 10 extinction sessions, administration of a small quantity of alcohol reinstated previously extinguished alcohol-seeking behavior. These results are consistent with the widely reported description of the “first-drink” phenomenon—that is, ingestion of a small alcohol amount may induce a strong subjective state of craving and, subsequently, relapse to drug-taking behavior in abstinent alcoholics (Ludwig et al. 1974). This priming effect can occur even after years of abstinence (Besancon 1993).

Recently, other research groups also have used the alcohol reinstatement paradigm. In those studies, stress caused by intermittent mild electric shocks to the animals’ feet (Lê et al. 1998) as well as alcohol-associated olfactory cues (Katner et al. 1999) could reinstate previously extinguished responding for alcohol. In conclusion, the characteristics of reinstatement of alcohol-seeking and -taking behavior are similar to those for other drugs of abuse. Furthermore, the reinstatement approach can be used to study the effects of putative anticraving and antirelapse medications.

Nevertheless, the usefulness of the reinstatement model in representing human alcohol dependence has two important limitations. First, researchers to date have not conclusively demonstrated that rats which go through a reinstatement procedure are truly alcohol dependent in the sense that they exhibit alcohol responding that is no longer controlled by normal behavioral mechanisms (i.e., is uncontrolled). Second, it appears that extinction of alcohol-seeking behavior usually plays only a minor role in alcoholic patients trying to achieve and maintain abstinence. With the exception of patients undergoing focused extinction therapy, alcoholics generally try to avoid exposure to external alcohol cues during abstinence. In most cases, alcoholics stay abstinent for a while but may experience craving and subsequent relapse if they are reexposed to external cues (e.g., the sight of a bar or smell of alcohol), particularly if they are in a vulnerable internal state. Consequently, the animal reinstatement procedure may not accurately reflect the situation of abstinent alcoholics experiencing craving and relapse. This situation may be better mimicked by the so-called alcohol deprivation effect (ADE), which is represented in an animal model in which long-term alcohol self-administration alternates with repeated alcohol deprivation phases.

## Long-Term Alcohol Self-Administration With Repeated Alcohol Deprivation Phases: An Animal Model of Alcoholism

To model the compulsive, uncontrolled alcohol-seeking and alcohol-taking behavior characteristic of human alcoholics, Spanagel and Hölter (1999) developed a long-term model of alcohol self-administration with repeated alcohol deprivation phases. In this model, male Wistar rats have free access to food, water, and three alcohol solutions of 5, 10, and 20 percent (volume/volume) in their cage. After two months of continuous alcohol access, the rats are deprived of alcohol for several days before again being offered all alcohol solutions. This procedure is repeated monthly for the following year. The renewed availability of the alcohol solutions following a deprivation phase leads to a pronounced but temporary rise in alcohol intake and preference, the ADE. This pattern of relapse-like drinking is observed across several species, including rats, mice, monkeys and human social drinkers (Sinclair 1971; Burish et al. 1981).

In addition to the ADE, alcohol consumption behavior after long-term consumption followed by deprivation also is characterized by changes in the animals’ alcohol intake patterns. Thus, the animals not only consume more alcohol but also consume large amounts of highly concentrated alcohol solutions at inappropriate times during their daily cycle (e.g., during the light phase when the animals are normally inactive and drinking activity is low).

Interestingly, the ADE in chronically drinking rats can persist over long abstinence periods (i.e., several months), demonstrating that a specific memory for the drug exists. This persistence is similar to the behavior of human alcoholics, who can easily relapse even after years of abstinence.

An ADE can also be observed under operant conditions—that is, if the animals have to perform a task to receive the alcohol. In these experiments, the animals’ alcohol intake and preference increase significantly following an alcohol deprivation phase of 2 weeks (Hölter et al. 1997), implying that a strong motivation exists for the drug. This strong motivation to drink a highly concentrated alcohol solution following deprivation is further demonstrated by the introduction of various progressive-ratio tasks, in which the animals have to work more and more (e.g., press a lever more often) in order to receive a reinforcer (e.g., alcohol). In such studies the maximum number of consecutive lever responses the animals will perform in order to receive one alcohol dose (i.e., the breaking point) is significantly higher following deprivation compared with baseline responding (Spanagel and Hölter 2000). These findings suggest that at least in chronically drinking rats, the ADE represents a situation of increased motivation to work for alcohol, which is compatible with the operational definition of craving (Markou et al. 1993).

Such increased motivation to work for alcohol, however, is not the primary criterion for defining addiction in animals—the loss of control over drinking also must be demonstrated. In an attempt to assess uncontrolled drinking behavior, researchers have sought to influence the ADE by either adulterating the taste of the alcohol solution with quinine or by offering a highly palatable sugar solution instead of water. In the first experiment, the investigators added quinine hydrochloride to the alcohol solution, but not to the water (Spanagel et al. 1996). Quinine is a very bitter tasting substance that usually produces a strong taste aversion in rats. Despite the aversive taste, however, the long-term alcohol-drinking rats consumed large amounts of the quinine-containing alcohol solution following a deprivation phase. In fact, alcohol intake and preference, as well as the time course of the ADE in the quinine-exposed animals, were similar to those of control animals that had the same experimental history and which received unadulterated alcohol. It is important to note, however, that increasing quinine concentrations did affect the expression of the ADE. Thus, when the alcohol was adulterated with high quinine concentrations, alcohol consumption and preference after deprivation dropped even below baseline drinking and preference. These results show that in long-term alcohol-drinking rats, alcohol intake following a deprivation phase is relatively resistant to modification by taste adulteration; in other words, drinking behavior to a certain point becomes inflexible and uncontrolled.

These conclusions were further supported by an experiment during which rats had a free choice between a sugar solution and alcohol after a period of alcohol deprivation (Spanagel and Hölter 1999). In general, rats have a high preference for the sugar solution over alcohol. Nevertheless, in this study, chronically drinking rats still consumed more alcohol following deprivation than before the deprivation period, indicating that the ADE was still present despite the availability of the sugar solution.

Thus, the two studies demonstrated that alcohol intake during the ADE remained unchanged after presentation of either an adulterated alcohol solution or a highly palatable sugar solution. These findings suggest that alcohol consumption in animals serves not only nutritional purposes but also is at least partly motivated by alcohol’s pharmacological effects. In other words, alcohol consumption during the ADE seems to involve compulsive, uncontrolled drug-seeking and drug-taking behavior and can clearly be dissociated from normal eating and drinking behaviors.

This conclusion is further supported by pronounced changes in the diurnal rhythm of drinking activity following alcohol deprivation in chronically drinking rats. For these experiments, the animals were tested in a fully automated electronic drinkometer device (Hölter et al. 1998) that allows researchers to monitor drinking patterns constantly on a computer. In the experiment, age-matched control animals exhibited normal drinking activity—that is, high drinking activity during the active night phase and low, and for some hours no, drinking activity during the inactive light phase. In contrast, the pattern of drinking activity changed in the chronically drinking rats during the ADE. In particular, most of the animals still showed high drinking activity during the inactive phase, and some animals even showed no differences in drinking activity during the dark and light phases of the daily cycle. Such a level of drinking activity is far beyond normal controlled behavior seen in the appropriate control animals.

In summary, the results of the alcohol deprivation studies indicate a nonnutritional component of alcohol consumption and pharmacologically motivated drinking behavior in long-term alcohol self-administering rats. Moreover, because the animals’ drinking behavior was difficult to modify, alcohol drinking during the ADE appears to represent compulsive, uncontrolled drug-seeking and drug-taking behavior. Additional studies demonstrated that chronically drinking rats that underwent repeated alcohol deprivation phases exhibited tolerance, physical and psychological signs of withdrawal, and stress-induced drinking (Hölter et al. 1998, 2000*b*; Spanagel and Hölter 2000). In particular, the animals showed augmented anxiety when experiencing alcohol deprivation, similar to the anxiety attacks observed in human alcoholics undergoing withdrawal. This experience of anxiety after alcohol deprivation also might contribute to the relapse-like drinking behavior observed in the rats.

Taken together these observations reflect some of the diagnostic criteria for alcoholism listed in the fourth edition of the *Diagnostic and Statistical Manual of Mental Disorders* (DSM–IV) of the American Psychiatric Association (1994). Thus, the alcohol deprivation model can serve as an animal model of alcoholism that covers most of the DSM–IV criteria. Any valid animal model of alcoholism, however, should have predictive value for the human situation. Therefore, anticraving drugs that can effectively prevent relapse in human alcoholics also should be effective in the animal model.

### Pharmacological Validation

#### Naltrexone

Numerous brain chemicals (i.e., neurotransmitters) help mediate alcohol’s pleasurable and reinforcing effects, including a group of molecules called endogenous opioids. Opioids and other neurotransmitters exert their activities by interacting with docking molecules (i.e., receptors) on the surfaces of certain nerve cells (i.e., neurons). Clinical studies have shown that an agent that blocks the actions of opioid receptors—the opioid receptor antagonist naltrexone—reduces alcohol consumption and relapse rates in human alcoholics (O’Malley et al. 1992; Volpicelli et al. 1992). To validate the alcohol deprivation model, which reflects craving and relapse, researchers investigated under which treatment conditions naltrexone could reduce the ADE in long-term alcohol-drinking rats.

To this end, the investigators tested naltrexone’s effects under two conditions: (1) when the agent was administered continuously and (2) when the animals received repeated intermittent naltrexone injections (Hölter and Spanagel 1999). Surprisingly, chronic naltrexone treatment did not reduce the ADE but enhanced alcohol preference. Conversely, intermittent injections of naltrexone moderately reduced the ADE. These opposing effects can be explained on a pharmacological level. Thus, chronic naltrexone administration permanently blocks the opiate receptors. To compensate for this blockade and maintain its normal level of opioid activity, the body produces more opioid receptors, thereby rendering the endogenous opioid system more sensitive to alcohol’s effects and enhancing alcohol preference. Conversely, intermittent naltrexone injections at moderate doses do not induce functionally relevant changes in opioid receptor levels, resulting in the predicted reduction of the ADE.

These findings emphasize the importance of selecting the right treatment regimen with naltrexone for obtaining a suppressant effect on drinking behavior and relapse. Thus, to maintain naltrexone’s effectiveness in blocking opioid receptors and reducing relapse drinking, one presumably needs a treatment regimen with a low dose and frequency of administration that prevents naltrexone accumulation. In light of these findings, it appears questionable whether naltrexone depots that continuously release the agent and, therefore, do not require the alcoholic to take the medication daily would be an appropriate treatment regimen.^3^ Furthermore, these findings suggest that low naltrexone doses might be more effective in selectively reducing alcohol craving and relapse than would be high doses.

#### Acamprosate

Another neurotransmitter mediating alcohol’s effects is glutamate, which interacts with several receptors, including the *N*-methyl-d-aspartate (NMDA) receptor. Even at low concentrations, alcohol may inhibit the activity of the NMDA receptor. An agent called acamprosate, which is a weak antagonist and modulator of the NMDA receptor, is used in most European countries to prevent craving and relapse (Sass et al. 1996). Acamprosate’s effects on relapse-like drinking also have been studied in the alcohol deprivation model. In these experiments, chronically drinking rats undergoing a deprivation phase received various acamprosate doses or a saline solution. Following alcohol representation, the animals received further acamprosate or saline injections. Compared with saline, acamprosate reduced alcohol intake and preference during the ADE in a dose-dependent manner (Spanagel et al. 1996). At the highest acamprosate doses, alcohol intake even dropped below baseline drinking.

Investigators also studied acamprosate’s effect on drinking behavior under operant conditions, both during normal training conditions (i.e., at baseline) and during the ADE (Hölter et al. 1997). Under baseline conditions, acamprosate reduced operant responding in the long-term alcohol-drinking rats. At maximal acamprosate levels in the blood and the brain, however, the agent reduced alcohol consumption more effectively during the ADE than during baseline drinking. Because the intensity of the ADE can serve as a measure of craving, these findings suggest that acamprosate indeed has anticraving properties.

The agreement between these findings and the clinical effectiveness of acamprosate and naltrexone in humans indicates that the alcohol deprivation model is a valid animal model in the search for new phamacotherapeutic agents for treating alcohol dependence. Hölter and colleagues (1996, 2000*a*) recently identified another NMDA receptor antagonist called memantine that effectively suppressed the ADE in chronically drinking rats. Future clinical trials must show whether this agent also is effective in humans as predicted by the animal model.

### Neurochemical and Molecular Changes During the ADE

In addition to allowing researchers to evaluate putative anticraving and anti-relapse compounds, the alcohol deprivation model may further improve understanding of the neurochemical and molecular mechanisms underlying addictive processes. To date, such analyses have focused primarily on the neurotransmitters γ-aminobutyric acid (GABA) and glutamate and their receptors. GABA is the primary inhibitory neurotransmitter—that is, it reduces the activity of the signal-receiving neuron—whereas glutamate is the primary excitatory neurotransmitter—that is, it stimulates the activity of the signal-receiving neuron. Alcohol has been shown to activate the GABA system and to inhibit the glutamate/NMDA receptor system.

In one study, researchers determined the accumulation of these neurotransmitters in and release from brain tissue slices of chronically drinking rats (Darstein et al. 1998). The study found no differences in GABA accumulation between alcohol-experienced and age-matched control rats. In contrast, NMDA receptor function was markedly enhanced in several brain regions (e.g., the striatum and nucleus accumbens—regions of the brain’s reward system that mediate the reinforcing properties of alcohol and other drugs of abuse) of the alcohol-drinking animals compared with the control animals. Furthermore, experiments revealed a selective increase in the production of various alcohol-sensitive components (i.e., subunits) of the NMDA receptor.^4^ A selective increase in the activity of the NMDA receptor system may explain the excessive excitability of the central nervous system during abstinence. Neuronal hyperexcitability, in turn, may trigger craving and relapse (Spanagel and Zieglgänsberger 1997). This hypothesis is supported by the finding discussed in the previous section that NMDA receptor antagonists such as memantine can dampen neuronal hyperexcitability and prevent the ADE (Hölter et al. 1996, 2000*a*).

Another study examined the integrity of the cytoskeleton—a system of fiber-like structures in the cell that serves to maintain the cell’s shape and stability—in long-term alcohol-drinking animals. The integrity of the cytoskeleton is a key factor for neuronal function, and changes in cytoskeletal properties correlate with certain aspects of the brain’s ability to adapt to various environmental and genetic influences (i.e., neuronal plasticity). The cytoskeleton consists of different fibrillar elements (i.e., microtubules) as well as various microtubule-associated proteins (MAPs). Studies in chronically drinking animals found that the amount of MAP2, which is a neuron-specific protein, was markedly reduced in these animals (Putzke et al. 1998). This effect was most pronounced in neuronal systems called the extrapyramidal system and the mesolimbic system, which are involved in motor control and motivation. These results suggest that long-term alcohol consumption may induce motor dysfunction and changes in motivational process by altering the integrity of the cytoskeleton.

## The Point-of-no-Return Model

Alcohol consumption patterns generally develop in several stages. Following a relatively short phase of acquisition of alcohol drinking behavior during which the individual experiments with different alcohol doses, a controlled alcohol drinking behavior generally develops. Under certain conditions, however, drinking behavior can become uncontrolled in some individuals. Researchers still do not know for certain whether a specific “point of no return” exists at which an irreversible “loss of control” occurs that is indicative of alcohol addiction. The concept of such a point of no return suggests the succession of two distinct states of alcohol consumption separated by a transition period (Coper et al. 1990).

To determine whether such a point of no return exists in the development of alcohol dependence, researchers have established an animal model on the development of “loss of control” (Wolffgramm 1991; Wolffgramm and Heyne 1995). In this model, rats are offered free access to water and to alcohol solutions with concentrations of 5, 10, and 20 percent. Under these conditions, alcohol-taking behavior initially seems to be exploratory. During this acquisition phase, days with high alcohol consumption alternate with days of near abstinence. Both the temporal drinking pattern and the daily dose are unstable and nearly unpredictable at this stage. Furthermore, the taste and odor of the alcohol solutions strongly influence the animal’s choice of drinking fluid. During this phase, which lasts 1 to 2 weeks, the rats learn to assess alcohol’s psychotropic effects and to adjust their intake behavior.

Subsequently, each rat develops an individual alcohol intake pattern that remains stable for several months (Wolffgramm 1990). During this phase, alcohol consumption is “controlled” by the interaction of both external and internal factors. For example, social behavior and social rank as well as such stressors as social isolation have a profound influence on drinking behavior (Wolffgramm 1990; Wolffgramm and Heyne 1991). In general, during this controlled phase the animals appear to use alcohol according to its psychotropic effects. Thus, dose and temporal consumption patterns are adjusted to the situation and to the individual rat’s internal state.

After approximately 6 months of continuous access to alcohol, the rats gradually change their alcohol-taking behavior. In contrast to the previous stable intake, they generally exhibit increasing alcohol consumption over the next few months, even though the environmental conditions remain constant (Wolffgramm and Heyne 1991).

In the first experiment using this model, the rats had access to alcohol for 9 months, followed by a long-term abstinence period of 9 months. After this period the rats again had access to the alcohol solutions. During this reexposure period, the animals exhibited a high preference for alcohol. This preference and the resulting high intake did not represent an ADE, however, because the ADE is a transient phenomenon that lasts only for a few days. In contrast, the animals reexposed to alcohol after prolonged abstinence exhibited high alcohol intake and preference over several weeks.

As already mentioned, addictive behavior is defined by a loss of control rather than just high alcohol intake. To test “loss of control” in this model, the investigators evaluated several factors that modify drinking behavior during controlled alcohol intake (i.e., in “non-addicted” animals). These factors included taste adulteration with quinine, exposure to a stressor (e.g., short-term isolation), and the individual dominance rank (Wolffgramm and Heyne 1991). Chronically drinking animals that had passed the stage of controlled drinking (i.e., “addicted” rats) and non-addicted rats were exposed to these factors during reexposure to alcohol. The results were as follows:

- Taste adulteration of the alcohol solution with quinine substantially reduced alcohol intake in nonaddicted rats. In contrast, addicted rats reduced their alcohol intake only to a lesser extent. In these animals, alcohol intake and preference remained substantially higher than in age-matched control rats without previous long-term alcohol experience.
- Short-term (i.e., 24 hours) isolation led to a considerable increase in alcohol intake in nonaddicted animals. This increase was not observed in addicted rats.
- Social rank influenced alcohol intake in nonaddicted rats; thus, subordinate rats consumed nearly twice as much alcohol as did dominant animals. In addicted rats, however, social rank no longer influenced alcohol intake and preference, and all animals exhibited the same pattern of alcohol consumption during reexposure to alcohol, regardless of social rank.

In summary, both external and internal factors that influence alcohol intake during controlled drinking lose their effect in animals that exhibit increasing alcohol intake after long-term alcohol exposure and abstinence. Furthermore, the transition from controlled to uncontrolled drinking appears to be irreversible in the rat, suggesting that a “point of no return” does indeed exist.

### Pharmacological Validation

The point-of-no-return model also has been validated pharmacologically using agents that modulate the activity of the neurotransmitter dopamine and its receptors (i.e., the dopaminergic system). A group of neurons called the mesolimbic dopaminergic system is thought to serve as a final common neural pathway for mediating reinforcement processes, and long-lasting neuroadaptive changes in this pathway develop during long-term use of alcohol and other drugs (Spanagel and Weiss 1999). Consequently, it appears likely that agents which interfere with dopaminergic neurotransmission might be useful as anticraving and antirelapse medications.

In one study, researchers tested the compound lisuride, which activates the D_2_ dopamine receptor (i.e., is a receptor agonist), in alcoholic patients as a putative anticraving and antirelapse compound (Schmidt et al. 1994). At the same time, lisuride was tested in alcohol-addicted and nonaddicted rats. In the animal model, lisuride treatment significantly increased alcohol intake in both addicted and nonaddicted animals, suggesting that the compound enhanced rather than reduced craving (May et al. 1995). Similar findings were obtained in the clinical study, in which lisuride-receiving patients were significantly more likely to relapse than were patients receiving an inactive control compound (i.e., a placebo).

Other investigators studied the effectiveness of the dopamine D_2_ receptor antagonist flupenthixol in preventing relapse both in alcoholic patients and in the loss-of-control model. As with the lisuride treatment, flupenthixol treatment enhanced alcohol intake in the animal model (Wolffgramm et al. 2000). In contrast to lisuride treatment, however, this effect was observed only in alcohol-addicted animals, but not in nonaddicted animals. The results of the clinical study confirmed the predictions made from the preclinical studies. Thus, flupenthixol-treated patients had a higher risk of relapse than did placebo-t reated patients (Wiesbeck et al. 2000). At the moment, it is unclear why both a dopamine receptor agonist and an antagonist produced a procraving effect in experimental animals as well as alcoholic patients. It is possible, however, that because lisuride and flupenthixol are not highly specific in binding to the dopamine D_2_ receptor, both agents also interact with other receptors in the brain that might play a role in this pro-craving effect. Nevertheless, these studies indicate that the point-of-no-return model can also predict the results of pharmacological interventions targeted at neurotransmitters in alcohol-addicted patients.

## Conclusions and Future Perspectives

This article has described three recent animal models used to investigate various aspects of alcoholism: (1) the alcohol reinstatement model, (2) the long-term model of alcohol self-administration with repeated alcohol deprivation phases, and (3) the point-of-no-return model. These models allow researchers to explore different dimensions of alcoholism compared with simple alcohol preference studies that only allow conclusions on the acquisition and maintenance of controlled alcohol intake behavior. Moreover, those conclusions do not substantially enhance understanding of the pathological processes underlying alcohol addiction and, therefore, are not appropriate for developing new relapse prevention strategies. Indeed, alcohol preference studies in animals often have led to conclusions that could not be transferred to the human situation. For example, preclinical alcohol preference studies in the past frequently were ineffective in predicting the results of new pharmacotherapies. However, the alcohol-preferring rat lines have been helpful for understanding the basic mechanisms underlying alcohol reinforcement. Moreover, researchers recently used alcohol-preferring P-rats in combination with the alcohol deprivation model, demonstrating that repeated alcohol deprivation phases led to a pronounced increase of voluntary alcohol consumption (Rodd-Henricks et al. 2000*a*).

The three newer models described here, which mimic aspects of craving, relapse, and “loss of control,” hopefully will open up new avenues in the pharmacological prevention of relapse. Furthermore, new molecular techniques, such as micro-DNA-array assays, that allow rapid genetic analyses of many individuals will enable researchers to conduct rigorous molecular analyses of animals derived from these models and to identify new genes that play a role in alcohol addiction. Despite the potential of these models, however, it is important to note that each model mimics only certain aspects of human addictive behavior. Consequently, further behavioral studies are warranted in order to understand the factors underlying these pathological conditions.

In addition, numerous sociocultural factors influence alcohol drinking in humans and therefore need to be considered in designing animal models. One extreme example of such influences is the drinking behavior in Scandinavian countries, which is frequently characterized by “Saturday night” intoxication. To be able to mimic such a behavior, animal models must be adapted to include the particular sociocultural situation. For example, Wahlström (1994) adapted an animal model to the characteristic Scandinavian drinking habits by once a week offering male rats a choice between alcohol and water before injecting them with a high alcohol dose. In this model, the animals developed a need for a stable daily alcohol dose but exhibited no loss of control under these conditions—that is, their alcohol consumption did not escalate and normal behavioral regulatory mechanisms still were effective. These findings suggest that sophisticated animal models can indeed represent complex human drinking behaviors, including the sociocultural factors that influence those behaviors.

## Footnotes

1 For many rodents, however, it is difficult to get them to drink substantial amounts of alcohol, because they find the taste aversive.

2 For a definition of this and other technical terms used in this article, see the central glossary, pp. 141–143.

3 Some clinical trials of these naltrexone depots have already been initiated.

4 This increase likely represents the body’s attempt to compensate for alcohol’s inhibition of NMDA receptor function, thereby maintaining normal NMDA receptor activity. In the absence of alcohol, however, the additional NMDA receptors may result in excessive NMDA receptor activity.
